# Complex effects of mammalian grazing on extramatrical mycelial biomass in the Scandes forest‐tundra ecotone

**DOI:** 10.1002/ece3.3657

**Published:** 2017-12-14

**Authors:** Tage Vowles, Frida Lindwall, Alf Ekblad, Mohammad Bahram, Brendan R. Furneaux, Martin Ryberg, Robert G. Björk

**Affiliations:** ^1^ Department of Earth Sciences University of Gothenburg Gothenburg Sweden; ^2^ Department of Biological and Environmental Sciences University of Gothenburg Gothenburg Sweden; ^3^ Terrestrial Ecology Department of Biology University of Copenhagen Copenhagen Denmark; ^4^ Center for Permafrost Department of Geoscience and Natural Resource Management University of Copenhagen Copenhagen Denmark; ^5^ School of Science and Technology Örebro University Örebro Sweden; ^6^ Department of Organismal Biology Uppsala University Uppsala Sweden; ^7^ Department of Botany Institute of Ecology and Earth Sciences University of Tartu Tartu Estonia; ^8^ Gothenburg Global Biodiversity Centre Gothenburg Sweden

**Keywords:** *Betula nana*, *Betula pubescens* subsp. *czerepanovii*, ectomycorrhiza, extramatrical mycelia, herbivory, mountain birch forest, shrub heath

## Abstract

Mycorrhizal associations are widespread in high‐latitude ecosystems and are potentially of great importance for global carbon dynamics. Although large herbivores play a key part in shaping subarctic plant communities, their impact on mycorrhizal dynamics is largely unknown. We measured extramatrical mycelial (EMM) biomass during one growing season in 16‐year‐old herbivore exclosures and unenclosed control plots (ambient), at three mountain birch forests and two shrub heath sites, in the Scandes forest‐tundra ecotone. We also used high‐throughput amplicon sequencing for taxonomic identification to investigate differences in fungal species composition. At the birch forest sites, EMM biomass was significantly higher in exclosures (1.36 ± 0.43 g C/m^2^) than in ambient conditions (0.66 ± 0.17 g C/m^2^) and was positively influenced by soil thawing degree‐days. At the shrub heath sites, there was no significant effect on EMM biomass (exclosures: 0.72 ± 0.09 g C/m^2^; ambient plots: 1.43 ± 0.94). However, EMM biomass was negatively related to *Betula nana* abundance, which was greater in exclosures, suggesting that grazing affected EMM biomass positively. We found no significant treatment effects on fungal diversity but the most abundant ectomycorrhizal lineage/cortinarius, showed a near‐significant positive effect of herbivore exclusion (*p* = .08), indicating that herbivory also affects fungal community composition. These results suggest that herbivory can influence fungal biomass in highly context‐dependent ways in subarctic ecosystems. Considering the importance of root‐associated fungi for ecosystem carbon balance, these findings could have far‐reaching implications.

## 1 INTRODUCTION

Ectomycorrhizal (ECM) associations are widespread in arctic and subarctic ecosystems (Newsham, Upson, & Read, 2009) and are potentially of great importance for carbon (C) and nutrient cycling (Averill, Turner, & Finzi, 2014; Clemmensen et al., 2013, 2015; Ekblad et al., 2013). Tundra plants have been found to divert up to 17% of their photosynthetic C to mycorrhizal fungi and in turn receive up to 61% of their required nitrogen (N) (Hobbie & Hobbie, 2006; Yano, Shaver, Giblin, & Rastetter, 2010). This plant–fungus mutualism has implications for C dynamics on a wide scale. For instance, a major part of the stored C in boreal soils is derived from roots and associated fungi, rather than aboveground plant litter (Clemmensen et al., 2013). Furthermore, on a global scale, soils in ecosystems dominated by ECM and ericoid mycorrhizal (ERM) host plants contain 70% more C per unit nitrogen than soil in ecosystems dominated by arbuscular mycorrhiza (AM)‐associated plants (Averill et al., 2014), underlining the importance of mycorrhizae as regulators of ecosystem C dynamics. Accordingly, factors influencing plant–fungus interactions, such as herbivory, may potentially have global implications.

Herbivores such as reindeer (*Rangifer tarandus*) and moose (*Alces alces*) play an important part in shaping arctic and subarctic plant communities and can substantially reduce the biomass of mountain birch, *Betula pubescens* subsp. *czerepanovii* (N. I. Orlova) Hämet‐Ahti (Kumpula, Stark, & Holand, 2011; Öhmark, Iason, & Palo, 2015), and dwarf birch, *Betula nana* L. (Kumpula et al., 2011; Olofsson et al., 2009; Post & Pedersen, 2008), which are both important host plant species for ECM fungi (Clemmensen, Michelsen, Jonasson, & Shaver, 2006; Michelsen, Schmidt, Jonasson, Quarmby, & Sleep, 1996; Ruotsalainen, Markkola, & Kozlov, 2009). Browsing of these host plant species is likely to also affect mycorrhizal fungi, and several studies show a negative effect of grazing on mycorrhizae (e.g., Markkola et al., 2004; Rossow, Bryant, & Kielland, 1997; Saravesi et al., 2015). The reason for this may be that the decline in photosynthate production impairs the plant's ability to supply its symbiont with C (Daft & Elgiahmi, 1978; Ekblad et al., 2013; Gehring & Whitham, 1991, 2002). However, the herbivore–plant–fungus interaction is complex, and in some ecosystems, an increase in mycorrhizal colonization in response to grazing has been found (Bayne, Brown, & Bethlenfalvay, 1984; Wallace, 1981), while a meta‐analysis of 99 studies found no significant decline in mycorrhizal colonization under herbivory (Barto & Rillig, 2010). One possible reason for these contradictory findings is that grazing affects not only mycorrhizal colonization but also species composition (Gehring & Whitham, 2002; Saikkonen et al., 1999). Mountain birch defoliation, for instance, may shift ECM fungal communities toward lineages with lower C requirements, which may hold a competitive advantage over those that invest in more extensive soil exploration when photosynthetic capacity is reduced (Parker et al., 2017; Saikkonen et al., 1999; Saravesi et al., 2015). In other words, it is possible that different fungal taxonomic groups differ in their response to a reduction in C supply (Gehring & Whitham, 1994).

ECM fungi produce extramatrical mycelia (EMM) that grow from ECM root tips into surrounding soil to forage for nutrients and seek new root tips for colonization (Anderson & Cairney, 2007). If grazers cause a shift in ECM species composition through favoring species of certain exploration types, grazing effects on EMM biomass should be particularly pronounced, but as most studies have focused on mycorrhizal colonization of roots, little is known about the effects on EMM (Ekblad et al., 2013; Gehring & Whitham, 2002). EMM play a key part in ecological processes such as plant nutrient uptake (Harley, 1989) and N cycling (Hodge & Fitter, 2010), and are likely to have an important role in C cycling (Cairney, 2012; Ekblad et al., 2013). Thus, there is a need to increase our understanding of the importance of herbivory on EMM production and community dynamics (Ekblad et al., 2013).

Using 16‐year‐old herbivore exclosures, we investigated the effects of grazing on EMM biomass during one growing season in two different vegetation types at a total of five sites in the Scandes forest‐tundra ecotone. We hypothesize that excluding large herbivores will positively influence EMM biomass, through an increased abundance of important ECM host plant species. Furthermore, we hypothesize that a long‐term release from grazing will also affect the fungal community composition, as competitive interactions will shift when disturbance is decreased.

## 2 MATERIALS AND METHODS

### 2.1 Study sites

The study was conducted over the growing season of 2011, in two southern study areas, Fulufjället (61°N, 12°E) and Långfjället (62°N, 12°E), and one northern area, Pulsuvuoma (68°N, 21°E), in the Scandinavian mountain range. The two southern study areas consisted of two sites each—a shrub heath (above the forest line) and a mountain birch forest site, whereas in Pulsuvuoma, there was a mountain birch forest site only. This made a total of five sites. The soils at the sites have podsol profiles throughout, with till and, in some cases, weathered soil at the base (Eriksson, Niva, & Caruso, 2007). The humus layer is about 3–5 cm.

Fulufjället is the southernmost field site. The shrub heath, located on a north‐facing slope at 930 m a.s.l., is characterized by the ECM‐forming *B. nana* in the shrub layer. The field layer, however, is dominated by ERM‐forming dwarf shrubs such as *Calluna vulgaris* (L.) Hull*, Vaccinium vitis‐idaea* L.*, Vaccinium myrtillus* L., and *Empetrum nigrum* subsp*. hermaphroditum* (Hagerup) Böcher along with the graminoid *Deschampsia flexuosa* L. In the bottom layer, lichens such as *Cladonia spp*. and *Cetraria islandica* (L.) Ach. are abundant. The birch forest site (880 m a.s.l.) lies in a low forest dominated by ECM‐forming *Betula pubescens* subsp*. czerepanovii* (from here on just *B. pubescens*) with an undergrowth of dwarf shrubs, such as *Vaccinium* spp. and *E. nigrum,* and grasses, such as *D. flexuosa* and *Nardus stricta,* as well as herbs, mainly *Trientalis europea* L. and *Solidago virgaurea* L. In the bottom layer, mosses such as *Pleurozium schreberi* L. and *Barbilophozia lycopodioides* (Wallr.) Loeske, are more common than lichens. Unlike at the other sites, reindeer husbandry has not been practiced at Fulufjället since the 19th century (Naturvårdsverket 2002).

The second study area, Långfjället, is also located in the southern part of the mountain range, about 55 km north of Fulufjället. The vegetation is similar to Fulufjället but the birch forest at Långfjället also contains scattered occurrences of *Pinus sylvestris* L. The shrub heath site (840 m a.s.l.) is located on an east‐facing slope and is grazed by reindeer from June to September, whereas the birch forest (800 m a.s.l.) is mainly grazed in June, before the reindeer are driven up to higher elevations by the emerging mosquitos, and in October, as the herds pass through on their way back down to the winter pastures (Jörgen Jonsson, Idre Sami Village, pers. comm.). Over the experimental period (1995–2011), the number of reindeer has been fairly constant with about 3.0 reindeer per km^2^ (Vowles et al., 2017).

At the Pulsuvuoma birch forest site (460 m a.s.l.), the tree layer is completely dominated by *B. pubescens*. The dominant species in the field and bottom layer are largely the same as at the southern sites, with the most noticeable difference being the absence of *C. vulgaris* (see Vowles et al. (2017) for full list of species abundances at all sites). The Pulsuvuoma site is frequented by reindeer approximately between November and January, although this varies considerably between years (Per‐Gustav Idivuoma, Lainiovuoma Sami village, pers. comm.). In contrast to Långfjället, the number of reindeer increased over the exclosure period (1995–2011) from about 2.0 to 3.5 reindeer per km^2^ (Vowles et al., 2017)

### 2.2 Experimental design

In 1995, six plots (25 × 25 m) were established at each site (see Eriksson et al. (2007) for full background), and the same plots were used in this study. Three of the plots at each site were surrounded by fences, 1.7 m high (hereafter referred to as exclosures). The fences exclude large herbivores, such as moose and reindeer, while small herbivores, such as hares and rodents, still had full access due to the large mesh size. The other three plots (from here on referred to as “ambient” plots, as they represent natural, i.e., grazed, conditions) were marked only with dug out corners and were left accessible to grazers.

### 2.3 EMM biomass

To estimate the effect of grazer exclusion on EMM biomass, four nylon sand‐filled ingrowth mesh bags (mesh size 50 μm; length 10 cm and diameter 2 cm; 40.0 g silica sand, free of organic matter; 550°C/24 hr) per plot were installed in May or June 2011 (see below), 3–4 m from the center of the plot in each cardinal direction. Immediately prior to the installation, a 2‐cm soil core, 10 cm long, was removed and each bag was vertically inserted such that the upper end was level with the soil surface (and left exposed). The sand‐filled bags are mainly colonized by mycorrhizal fungi due to the absence of a carbon source (Wallander, Ekblad, & Bergh, 2011). In fall 2011, just before the soil froze, the bags were collected and frozen within a few hours before further processing. The incubation time per site was 148 (24 May – 20 Oct), 148 (25 May – 21 Oct), and 112 (8 June – 27 Sept) days for Fulufjället, Långfjället, and Pulsuvuoma, respectively.

The sand from the four bags from each plot (in total 160 g dry weight) was mixed, and fungal mycelia were extracted from a subsample of 80.0 g wet weight using the technique of Kjöller et al. (2012). The extracted mycelia samples were then freeze‐dried. When dry, the extracted mycelia were further rinsed from sand grains before analyzed for C and N concentrations. Some sand particles still adhered to the harvested mycelia, and the N% data were therefore corrected assuming a C concentration of 49.75%, which was the highest C% found and taken to represent sand‐free mycelia. To obtain the water content of the sand, the remaining sand sample was freeze‐dried and then weighed allowing for correction of the biomass estimation.

### 2.4 Temperature measurements

Temperature loggers (Tinytag plus 2 TGP‐4020, Gemini Data Loggers, UK) were installed at the same time as the mesh bags and soil temperature were recorded and logged once per hour in the center of each plot, at a depth of 2 cm, for the duration of the experiment. From the temperature data, thawing degree‐days (TDD), which is the sum of all mean daily temperatures above 0°C, were calculated according to Molau and Mølgaard (1996) for the period that the bags were in the ground.

### 2.5 Vegetation inventories

During the summer of 2011, vegetation inventories of each plot were carried out, in order to quantify abundances of the dominant ECM‐forming species (*B. pubescens* at the birch forest sites and *B. nana* at the shrub heath sites). For the tree layer inventories in the birch forest, a 1.5 m strip along the edges was excluded, to avoid edge effects, and the remaining 22 × 22 m area of each plot was divided into 16 squares of 5.5 × 5.5 m each. In six randomly selected squares of the 16, we measured height and base diameter for every birch taller than 20 cm. For trees taller than 130 cm, diameter at breast height was also measured. The biomass (dry weight of living tree tissue) of the mountain birch stands in each plot was then estimated using the equation developed by Dahlberg, Berge, Petersson, and Vencatasawmy (2004):

$$\text{Tree biomass per plot}\;\left(\text{kg}\right)=\sum_{i=1}^{n}e^{\left(-5.4923+0.9803*\text{Ln}\left({\text{TBA}}_{i}\right)\right)}$$

TBA_*i*_ is the cross‐sectional area per tree at breast height (mm^2^), and n is the number of trees measured in the plot. The birch biomass per plot was then rescaled to kilograms per hectare. As the equation uses area at breast height, only individuals taller than 130 cm were included in biomass estimations. Unfortunately, tree inventories could only be carried out in five of the six plots at the Fulufjället birch forest site.

For the field layer (i.e., herbs and small shrubs, excluding cryptogams) inventories, a 1.5 m strip was again excluded from each plot, and the remaining area was divided into 484 1 × 1 m subplots. Twenty of these 1 × 1 m subplots were randomly chosen and a 0.5 × 0.5 m steel grid, divided into 100 equally sized quadrats, was laid out in the southern corner of each subplot. Frequencies (cover) of all species were then recorded by counting the number of quadrats in which each species occurred.

We then used *B. pubescens* biomass and *B. nana* frequency (for which no reliable function to estimate biomass was found in the literature) as factors in the linear models.

### 2.6 Soil and root sampling and analysis

Two soil cores were taken (and pooled) from each of two randomly selected subplots, using a soil core sampler (diameter 7 cm, length 10 cm). These were stored at 4°C until we returned to the laboratory, where they were frozen until analysis. Organic and mineral layers were separated out and sieved through a 2 mm mesh. Subsamples from both layers were analyzed for C and N content through mass spectrometry (ANCA‐TGII interfaced with a 20–20 IRMS, SerCon, UK), as well as pH and loss on ignition. Roots <2 mm diameter from both soil horizons were also extracted and analyzed, separately, through mass spectrometry.

### 2.7 DNA extraction, amplification, and sequencing

After the sand from the four bags from each plot was pooled, DNA was extracted from 500 mg (dry weight) subsamples using a NucleoSpin Soil kit (Machery‐Nagel GmbH, Germany). The SL1 lysis buffer and SX enhancer included in the kit were used. DNA was eluted in 20 μL of the supplied elution buffer. DNA was quantified in the extracts by fluorometry (QuBit 3.0, Thermo Fisher Scientific, USA), with individual extracts ranging from 4.32 to 24.6 ng total DNA. The second internal transcribed spacer (ITS2) region of ribosomal DNA was amplified by polymerase chain reaction (PCR) using the fungi‐specific gITS7 forward primer (Ihrmark et al., 2012) and the general eukaryote ITS4 reverse primer (White, Bruns, Lee, & Taylor, 1990). The gITS7 primer included unique indexed tags for each sample to allow for multiplex sequencing. Amplication of the ITS2 region was performed using a real‐time (quantitative) PCR instrument (CFX96 Touch, Bio‐Rad Laboratories, Inc., USA) in order to observe the process of the reaction and stop it, while the majority of the samples were still in the log‐linear amplification phase, as described by Urbina, Scofield, Cafaro, and Rosling (2016). The reaction mix was SSOAdvanced SYBR Green Master Mix (Bio‐Rad Laboratories, Inc., USA) at 1× concentration, with 0.8 μM of each primer and approximately 600 pg of extracted DNA. The PCR was run with the protocol: 98°C for 3 min, followed by 30 cycles of 98°C for 15 s, 52°C for 45 s, 60°C for 30 s, and finally a melt curve was generated from 65° to 95° in increments of 0.5°C. PCR products were cleaned using the ZR‐96 DNA Clean & Concentrator‐5 kit (Zymo Research Corporation, USA). DNA concentration was once again quantified by fluorescence using the QuBit 3.0 fluorometer, and up to 30 ng of each duplicate sample, if available, was pooled for sequencing. The pooled library was sequenced using a PacBio RS II (Pacific Biosciences, USA) at the Uppsala Genome Center (Uppsala University, Sweden) using four single‐molecule real‐time (SMRT) cells.

### 2.8 Bioinformatics

We used Mothur (v.1.34.4; (Schloss et al., 2009) for quality filtering and demultiplexing of the PacBIO consensus sequences (bdiffs = 1, minlength = 80, qwindowaverage = 30, qwindowsize = 50). Quality filtering was followed by demultiplexing as well as removing tags and primers using the command “trim.seqs” in Mothur with the following options: maxambig = 0, bdiffs = 1, minlength = 80, processors = 1, qwindowaverage = 30, qwindowsize = 50. ITS regions were extracted from quality‐filtered sequences using ITS extractor (Bengtsson‐Palme et al., 2013). For clustering of sequences, we used USEARCH (v8.0) (Edgar, 2010) using the command “cluster_fast” with standard options. Chimeras were detected using Uchime (Edgar, Haas, Clemente, Quince, & Knight, 2011) which along with sequences shorter than 100 bp long were discarded. The remaining sequences were used to generate operational taxonomic units (OTUs) based on 97% similarity threshold in Usearch (Edgar, 2010). OTUs were assigned to guild and lineage using default settings in FUNGuild (Nguyen et al., 2016) and hyphal exploration type according to the classification of Agerer (2006). Taxonomy and lineage were assigned based on the taxonomic information of the best blast hit. Lineages are designated by a slash (/) followed by the dominant genus, genera, or higher level taxon. Blast searching was performed against UNITE database (Koljalg et al., 2013). Relative abundances of guilds, lineages, and exploration types were calculated for each plot.

### 2.9 Data analyses

All statistical analyses were carried out using the software R (R Core Team 2012). Linear models were used to test for differences in EMM biomass. We analyzed the birch forest and shrub heath sites separately as we hypothesized that the abundance of the dominant ECM host plants in each plant community, which was *B. pubescens* at the birch forest sites and *B. nana* at the dry heath sites, is one of the main drivers of EMM production, and we wanted to include these measurements, along with soil TDD, as covariates in the models. Thus, the birch forest model included the factors treatment, site, *B. pubescens* biomass and soil TDD, whereas the shrub heath model used the factors treatment, site, *B. nana* frequency and soil TDD. Furthermore, we used separate linear models to evaluate whether there were any differences in *B. pubescens* biomass, *B. nana* frequency, and soil TDD between sites or treatments (i.e., with factors treatment*site) and then used the lsmeans package (Lenth, 2016) to obtain *t*‐ratios and *p*‐values of treatment effects at each site. All data were log‐transformed to fulfill assumptions of normality. In addition, we used linear regression to explore relationships between mycelial biomass and soil TDD and *B. nana* frequency.

We also used mycorrhizal fungal OTU data to calculate diversity indices for the fungal community, for both all mycorrhizal species and ECM species only. We calculated Shannon's H, Fischer's α, and the complement (1‐D) and reciprocal (1/D) forms of Simpson's D using the diversity function in the vegan package of R (Oksanen et al., 2013) and evenness indices of Simpson's D and Shannon's H according to Magurran (2004). Linear models were used to test for treatment differences in relative abundances of guilds, lineages, and exploration types as well as diversity measures, where, in contrast to the mycelia biomass models, both vegetation types were included in the model to increase statistical power.

We tested the effect of grazing, vegetation type, and soil variables, including pH, soil organic matter, and C:N ratio, on both ECM and total OTU composition using the multivariate version of ANOVA (PERMANOVA) as implemented in the adonis function of the vegan package of R (Oksanen et al., 2013). Each variable was tested in a single‐factor analysis. All results were considered significant if *p* < .05.

## 3 RESULTS

### 3.1 Mycelial biomass

**Figure 1:**
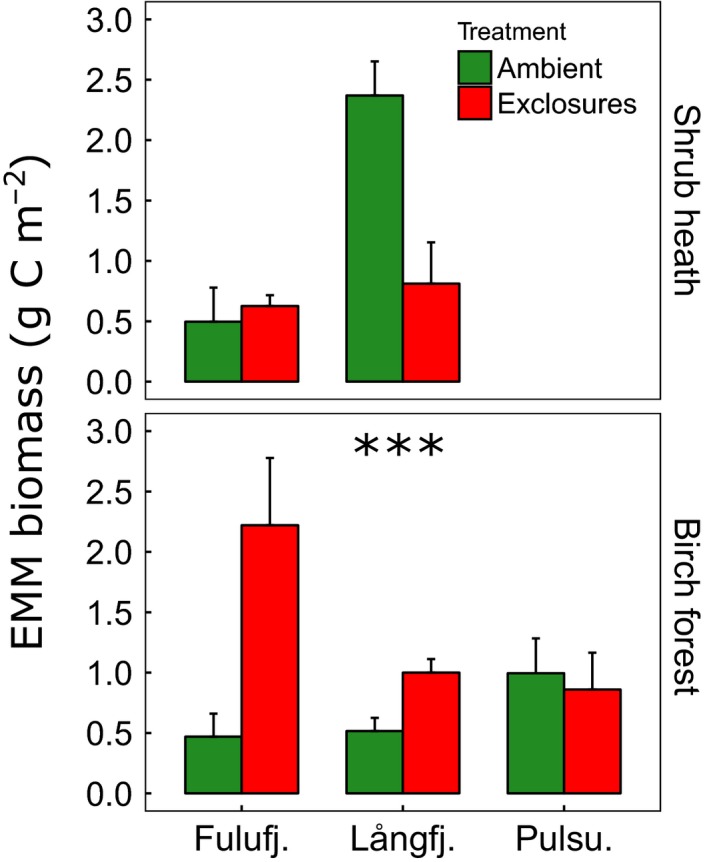
Mean extramatrical mycelial (EMM) biomass (±standard error, *n* = 3) at the five study sites. Grazer exclosure was significant at the birch forest sites (***p < .01). Ambient plots (green) were accessible to all herbivores, while exclosures (red) were surrounded by fences that keep out large mammals, such as moose and reindeer

**Figure 2:**
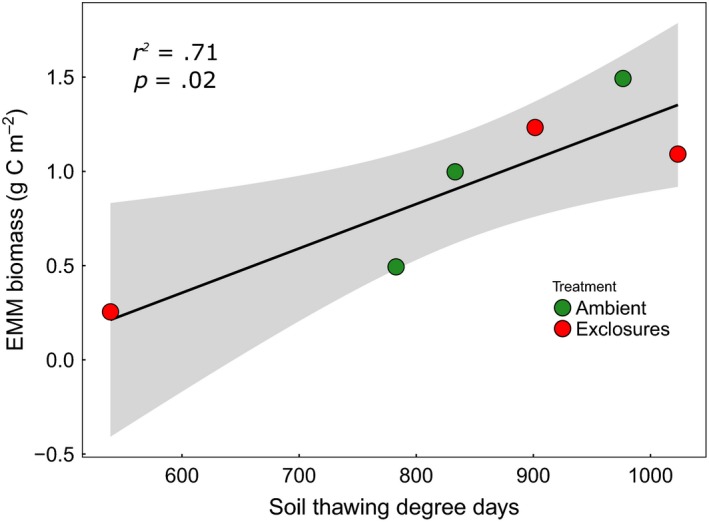
Relationship between extramatrical mycelial (EMM) biomass and soil thawing degree‐days at Pulsuvuoma birch forest site, including *R* ^2^ and *p*‐value from linear regression. Gray area shows 95% confidence interval. Ambient plots (green) were accessible to all herbivores, while exclosures (red) were surrounded by fences that keep out large mammals, such as moose and reindeer

In the birch forest at Fulufjället, mycelial biomass was more than four times as high in exclosures, and at Långfjället, it was nearly twice as high as in ambient plots (Figure 1). At Pulsuvuoma, there appeared to be no treatment effect, but overall the negative effect of grazing on mycelial ingrowth was significant (*p *< .01, Table 1). The effect of *B. pubescens* biomass on EMM biomass was not significant, but the effect of soil TDD was (*p* < .01). This appeared to be driven by a significant positive correlation between TDD and EMM biomass in Pulsuvuoma (Figure 2, Table 1).

**Table 1:** ANOVA results showing effects of treatment, site, *Betula pubescens* subsp. *czerepanovii* biomass, and soil thawing degree‐days (TDD) on extramatrical mycelia biomass at the birch forest sites

Birch forest	Df	*F*	*p*
Treatment	1	14.4	**.00**
Site	2	2.1	.18
*B. pubescens* biomass	1	0.1	.77
Soil TDD	1	12.1	**.01**
Treatment*Site	2	9.4	**.01**

Bold values significant at *p* < .05

**Figure 3:**
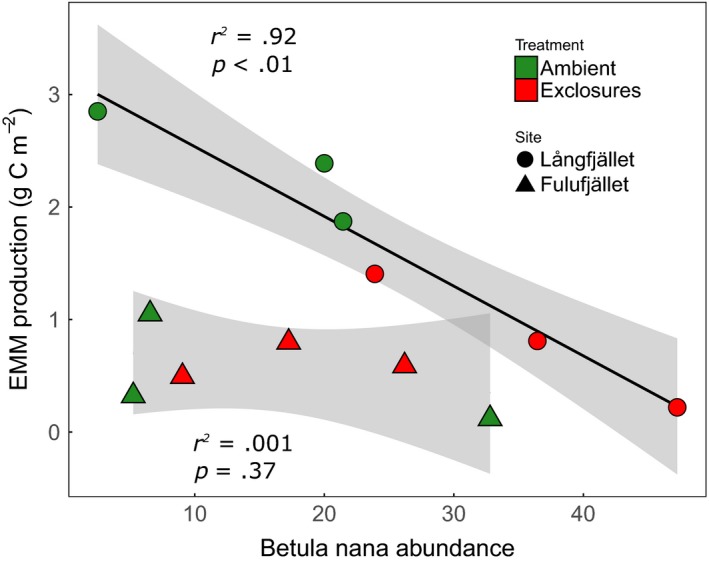
Relationship between extramatrical mycelial (EMM) biomass and Betula nana abundance (expressed as frequency between 1 and 100) at Fulufjället (triangles) and Långfjället (circles) shrub heath sites. Linear regression was significant at Långfjället. Gray areas show 95% confidence intervals. Ambient plots (green) were accessible to all herbivores, while exclosures (red) were surrounded by fences that keep out large mammals, such as moose and reindeer

At the shrub heath sites, the ambient plots at Långfjället had a threefold higher EMM biomass than exclosures, but at Fulufjället, where there are no reindeer, there was no difference (Figure 1). Overall, treatment was not statistically significant, although there was a significant site effect (*p *= .01, Table 2). There was also a near‐significant effect of *B. nana* frequency (*p* = .06), which at Långfjället was negatively correlated to mycelial biomass (Figure 3, Table 2).

**Table 2:** ANOVA results showing effects of treatment, site, *Betula nana* frequency, and soil thawing degree‐days (TDD) on extramatrical mycelia biomass at the shrub heath sites

Shrub heath	Df	*F*	*p*
Treatment	1	4.1	.09
Site	1	11.7	**.01**
*B. nana* frequency	1	5.6	.06
Soil TDD	1	2.3	.18
Treatment*Site	1	3.6	.11

Bold values significant at *p* < .05

### 3.2 Betula pubescens biomass, *B. nana* frequency, and soil TDD

We found no significant differences in *B. pubescens* biomass and *B. nana* frequency between exclosures and ambient plots, even though there was a near‐significant treatment effect on *B. pubescens* biomass at the Fulufjället birch forest site (*p* = .07). Soil TDD were significantly higher in exclosures at Fulufjället shrub heath and significantly lower in exclosures at Långfjället shrub heath (Table 3).

**Table 3:** Elevation, bedrock, soil type coordinates, and duration time of experiment at the different sites (additional data from Eriksson et al. (2007)) and differences in *B. nana* frequency, *B. pubescens* biomass and soil TDD. Bold values statistically significant at *p* < .05

	Shrub heath		Birch forest		
	Fulufjället	Långfjället	Fulufjället	Långfjället	Pulsuvuoma
Elevation (m a.s.l.)	930	840	880	800	460
Bedrock	Sandstone	Dala granite	Sandstone	Dala granite	Metagranodiorite, Metatonalite
Soil type	Gravelly till	Gravelly till	Gravelly till	Gravelly till	Till
Coordinates	61°38′11″ N	62°06′53″ N	61°38′45″ N	62°03′59″ N	68°20′19″ N
	12°38′29″ E	12°16′30″ E	12°35′34″ E	12°14′56″ E	21°19′35″ E
Duration of experiment	24 May – 20 Oct	25 May – 21 Oct	24 May – 20 Oct	25 May – 21 Oct	8 June – 27 Sept
Incubation time (days)	148	148	148	148	112
*B. nana* frequency (0–100)					
Exclosures	17.5 (±5.0)	35.9 (±6.7)	–	–	–
Ambient	14.9 (±9.0)	14.7 (±6.1)	–	–	–
*B. pubescens* biomass (kg/ha²)					
Exclosures	–	–	13,904 (±3188)	22,650 (±5732)	32,138 (±6131)
Ambient	–	–	7359 (±4559)	25061 (±2680)	35,218 (±3748)
TDD Soil					
Exclosures	**1270.3 (±18.2)**	**1198.3 (±11.4)**	1317.5 (±41.9)	1244.6 (±62.3)	821.2 (±145.5)
Ambient	**1209.9 (±13.8)**	**1298.0 (±24.7)**	1379.2 (±38.6)	1280.9 (±19.5)	864.2 (±58.0)

### 3.3 Fungal community composition

From 117,583 consensus sequences, 84,811 reads passed quality filtering. In total, of 84,811 quality‐filtered reads submitted to ITS extractor (Bengtsson‐Palme et al., 2013), 77,095 ITS2 regions were detected, of which 13 were chimeric. In addition, 100 and 44 were detected as reference‐ and de novo‐based chimeras, and 3,259 reads were less than 100 bp long. The average length of OTU sequences was 279.006 bp.

The remaining sequences were clustered into 1,353 OTUs. Of these, 851 were Ascomycetes, 306 Basidiomycetes, 15 Rozellomycetes, seven Zygomycetes, three Chytridiomycetes, one Glomeromycete, and the rest (170) were unidentified. As for functional guilds, 44 OTUs were classified as ERM species (which corresponds to an average relative abundance of 3%), 38 as ECM (average relative abundance 1%), one as AM (rel. abun. <1%), and 272 as saprotrophs (rel. abun. 22%). A total of 877 OTUs were of unknown functional guild (65%). Remaining OTUs (121) consisted of different endophytes, pathogens, and parasites (8%). Manual checking of the 20 most common OTUs returned as “of unidentified functional guild” in FUNGuild, which made up ~50% of all unidentified OTU sequences, revealed six additional saprotrophs but no mycorrhizal species.

**Figure 4:**
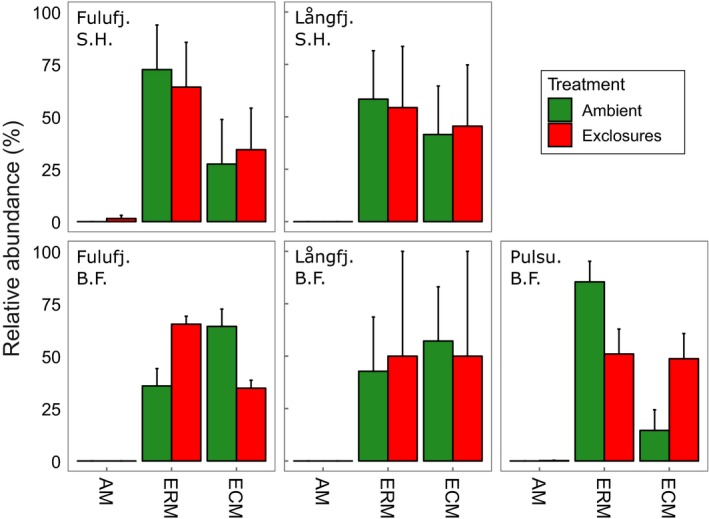
Relative abundances (mean ± SE) of arbuscular (AM), ericoid (ERM), and ectomycorrhiza (ECM) based on 83 identified mycorrhizal OTUs (after saprotrophs and unknowns had been filtered out). Ambient plots (green) were accessible to all herbivores, while exclosures (red) were surrounded by fences that keep out large mammals, such as moose and reindeer. Långfj, Långfjället; Fulufj, Fulufjället; Pulsu, Pulsuvuoma; S.H., Shrub heath; B.F., Birch forest

**Figure 5:**
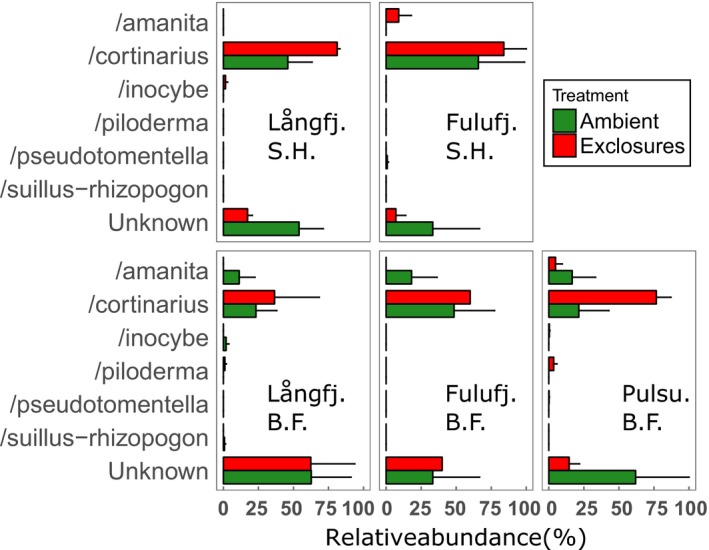
Relative abundances (mean ± SE) of ectomycorrhizal lineages based on 38 identified ectomycorrhizal OTUs. Ambient plots (green) were accessible to all herbivores, while exclosures (red) were surrounded by fences that keep out large mammals, such as moose and reindeer. Långfj, Långfjället; Fulufj, Fulufjället; Pulsu, Pulsuvuoma; S.H., Shrub heath; B.F., Birch forest

In total, ERM comprised a larger part of the identified OTUs than ECM, but we found no significant differences in relative proportions of AM, ERM, or ECM fungi between treatments, vegetation types, or sites (Figure 4). Nor could we find any significant differences in mycorrhizal species evenness. Of the identified ECM lineages, /cortinarius was the most abundant and was more abundant inside exclosures at all five sites (Figure 5), which was close to statistically significant (*p* = .08, Table 4). Because /cortinarius was so much more abundant than the remaining lineages, classification by hyphal exploration type yielded very similar results, with the medium‐distance fringe type, in our study represented by /cortinarius and /piloderma, showing a near‐significant effect of treatment (*p* = .07, Table 5).

**Table 4:** ANOVA results showing effects of treatment and site on identified ectomycorrhizal lineages as well as hyphal exploration type as classified by Agerer (2006) and Di Marino, Koljalg, and Agerer (2007). C, contact; SD, short‐distance; MDS, medium‐distance smooth; MDF, medium‐distance fringe; LD, long‐distance

Lineage	Exploration type	Treatment			Site		
		Df	*F*	*p*	Df	*F*	*p*
/amanita	C/SD/MDS^a^	1	1.13	.30	4	0.60	.67
/cortinarius	MDF (rarely SD)¹	1	3.36	.08	4	1.32	.30
/inocybe	SD (C/MDF) a	1	0.01	.93	4	0.46	.76
/piloderma	SD/MDF^a^	1	3.49	.08	4	1.40	.27
/pseudotomentella	MDS^b^	1	0.43	.52	4	0.91	.48
/suillus–rhizopogon	LD¹	1	0.83	.37	4	0.86	.51
Unknown		1	1.65	.21	4	0.86	.50

a Agerer (2006)

b Di Marino et al. (2007).

**Table 5:** ANOVA results showing effects of treatment and site on hyphal exploration type of ectomycorrhizal lineages as classified by Agerer (2006) and Di Marino et al. (2007)

Exploration type	Treatment			Site		
	Df	*F*	*p*	Df	*F*	*p*
Medium‐distance fringe/short‐distance	1	3.60	.07	4	1.20	.34
Contact/short‐distance/medium‐distance smooth	1	1.18	.29	4	0.60	.67
Long‐distance	1	0.83	.37	4	0.86	.51

The PERMANOVA showed no significant effect of treatment or any of the measured soil or root variables (Tables S1 andS2) on OTU community composition (*p* > .05).

## 4 DISCUSSION

We found that grazer exclusion had a significant positive effect on EMM biomass at the birch forest sites, where mycelial biomass was substantially higher in exclosures at both Fulufjället and Långfjället. The fact that the difference in mycelial biomass was greatest at Fulufjället, where there are no reindeer, suggests that moose browsing may have a greater impact on mountain birch forests than browsing by reindeer, which tend to not spend the summer months there (Bernes, Bråthen, Forbes, Speed, & Moen, 2015). Although no sufficiently fine‐scale moose population data are available from our study areas, Fulufjället is well known for its sizable moose population (Naturvårdsverket 2002), and numerous moose droppings were observed around the study site (T. Vowles, pers. obs.). Moose can decrease mycorrhizal colonization in taiga ecosystems through winter browsing of willow and poplar twigs (Rossow et al., 1997), and, furthermore, browse the sites throughout the summer, which would have a greater impact on the flow of photosynthates to roots and, subsequently, mycorrhiza, as ECM fungi are dependent on newly fixed carbon (Ekblad et al., 2013; Högberg et al., 2001). Contrary to our hypothesis, however, we found no correlation between *B. pubescens* biomass and EMM biomass which could mean that other factors connected to herbivory, such as trampling and fertilization, may have influenced mycelial production, although no correlation to any of the measured soil variables corroborated a fertilization effect either. Trampling from deer is known to sometimes cause soil compaction (Heckel, Bourg, McShea, & Kalisz, 2010; Shelton, Henning, Schultz, & Clay, 2014), which may affect mycorrhiza negatively (Entry, Rygiewicz, Watrud, & Donnelly, 2002), but as we did not measure soil porosity, we cannot make any such deductions from our study. Hence, although it is likely that the differences in EMM biomass in exclosures and ambient plots reflect herbivore influence, further research into the precise mechanisms behind this is needed.

Pulsuvuoma is the only site where EMM biomass was significantly related to soil TDD. No effect of herbivore exclosure was found, but considering that the site is generally only used by reindeer in winter, when they do not browse mountain birch and that moose are very rare in the area (P‐G Idivuoma, Lainiovuoma Sami village, pers. comm.), this is not surprising. Pulsuvuoma is located in the discontinuous permafrost zone (Gisnås et al., 2016), in contrast to Fulufjället and Långfjället that have no permafrost. Even though it has a shorter growing season, TDD vary more between plots at Pulsuvuoma than at the other sites. This suggests that underlying permafrost in places substantially influences the soil temperature. It has been shown that ECM fungi are able to survive subzero temperatures (Tibbett, Sanders, & Cairney, 2002), and there are indications that EMM in the arctic can be perennial (Clemmensen et al., 2006; Tibbett et al., 2002). However, the optimum temperature for ECM fungal growth is in general over +20°C for both arctic and temperate strains and at temperatures of 2°C hyphal growth slows down considerably (Tibbett, Sanders, & Cairney, 1998). In fact, arctic strains of the ECM *Hebeloma* fungi have been found to reduce their growth rate more than temperate strains at low temperatures, likely as a physiological adaptation to cold where resources in arctic strains are more readily diverted into carbohydrate buildup for cryoprotection (Tibbett et al., 1998). This slowed growth rate at low temperatures would explain why we found lower EMM biomass in the plots with the lowest number of TDD.

Surprisingly, at the shrub heath at Långfjället, EMM biomass was negatively related to the abundance of the dominant ECM host species, which at the shrub heath sites is *B. nana*. This contrasts with the finding that EMM biomass increases as *B. nana* production is stimulated through warming and fertilization (Clemmensen et al., 2006; Deslippe, Hartmann, Mohn, & Simard, 2011). In our study, however, the relative increase in *B. nana* cover was caused by a cessation of grazing (Vowles et al., 2017). It is possible that browsing and removal of *B. nana* biomass in ambient plots at the beginning of summer cause dieback and subsequent regrowth of EMM hyphae and that this increased mycelial turnover is the reason that we found more EMM biomass in grazed plots than in exclosures. In *P. sylvestris* forests, mycelia turnover time has been found to be higher in younger than older forest stands, possibly due to total belowground C flux being lower and N immobilization being higher in older forest stands (Hagenbo et al., 2017). The same could perhaps be true of exclosures compared to ambient plots at the Långfjället site, where in the absence of grazing, *B. nana* was allowed to proliferate, increasing the competition for resources. It could also have to do with the fact that *B. nana* is able to allocate large proportions of its biomass belowground and can translocate substantial amounts of nutrients between plant parts during the season (Chapin, Johnson, & McKendrick, 1980). This high plasticity helps it to respond quickly to altered environmental conditions (Bret‐Harte et al., 2001). Therefore, when *B. nana* is suppressed by grazers, it is possible that comparatively more C is allocated to the roots, which may stimulate fungal growth. Many plants are known to reallocate photosynthetic C from foliar tissue to roots in response to grazing, thereby protecting resources which may be stored and used for regrowth or to increase nutrient uptake (Babst et al., 2005; Holland, Cheng, & Crossley, 1996; Schwachtje et al., 2006). This may be the reason that mycorrhizal colonization of perennial grasses and forbs in some cases has been found to actually increase following herbivory (Barto & Rillig, 2010). It has been suggested that the increase in C exudates under grazing pressure, and subsequently in microbial activity and N mineralization, may select for maintaining high mycorrhizal colonization even under heavy defoliation, in order to facilitate plant access to the short‐term pulse of mineralized N following foliage removal (Barto & Rillig, 2010). Hence, it is possible that the larger EMM biomass in ambient plots compared to exclosures is a result of a larger allocation of C to belowground tissue, in order to optimize nutrient uptake in nutrient‐poor environments even when photosynthetic ability is impaired.

Although a previous study found that mountain birch defoliation caused by autumnal and winter moth (*Epirrita autumnata* and *Operophtera brumata*) altered ECM fungal communities (Parker et al., 2017), linear models in our study showed no significant treatment effects on diversity or evenness. Nor did we find any significant differences in the relative proportions of AM, ERM, and ECM fungi, despite the surprising finding that there were more ERM OTUs than ECM, which we assume were mostly spores due to wash offs, which would contribute little to biomass. We did, however, find indications that the most abundant lineage, /cortinarius, was positively affected by the exclusion of herbivores. One reason for this could be that *Cortinarius* spp., which can be sensitive to fertilization (Brandrud, 1995) and disturbance (Sun et al., 2015), benefit from the absence of trampling and fertilization through droppings and urine. It is also conceivable that a greater competition for resources caused by a release from grazing would favor nutrient uptake efficient yet C‐demanding mycorrhizal species such as *Cortinarius* spp., which have high‐biomass growth forms, medium‐distance exploration types, and the capacity to access limiting N by breaking down complex organic matter (Simard et al., 2015). However, further studies, with larger sample sizes, are evidently needed to delve deeper into the effects of grazing on fungal community dynamics.

The large abundance of /cortinarius is in line with other recent studies from the Scandes forest‐tundra ecotone (Parker et al., 2017; Saravesi et al., 2015), which nuances the view that *Cortinarius* spp. are unwilling to colonize mineral substrates (Wallander et al., 2013). Moreover, we cannot rule out that the sand substrate causes some kind of selection bias toward certain fungal species. Hendricks, Mitchell, Kuehn, Pecot, and Sims (2006) found that mycelial ingrowth was greater when natural soil was used as substrate rather than sand, which means that we may have consistently underestimated the mycelia production in our study. The use of natural soil as a substrate can also be problematical, however, due to the large and variable amounts of background fungal biomass (Wallander et al., 2013). Not much is known, to our knowledge, about mesh bag selection bias toward certain species, especially in tundra areas and is a question that needs to be investigated further. Sequencing of sampled soil cores adjacent to the mesh bags could in future studies give valuable information about resident fungal communities, even though problems relating to turnover times may arise in such comparisons. Another surprising finding was that ERM comprised a larger part of the identified OTUs than ECM. How much ericoid mycorrhizas contribute to the soil mycelium is largely unknown (Read & Perez‐Moreno, 2003), but in boreal and temperate forests, EMM is thought to be mainly produced by ECM fungi associated with trees (Ekblad et al., 2013). It is possible that in ericoid‐dominated ecosystems like ours, ERM produces more EMM than previously recognized and is a question that is in need of further research.

On a final note, our results showed that the EMM biomass in the studied subarctic vegetation types is low compared to those reported previously in boreal and temperate forests (Ekblad et al., 2013). The average ambient EMM biomass recorded was 0.66 g C/m^2^ in the birch forest and 1.43 g C/m^2^ on the shrub heath. There was considerable variation between sites, especially at the shrub heath where the mycelial biomass in Långfjället was nearly five times as high as in Fulufjället. Despite this, the results are comparable with earlier findings from a dry subarctic heath in Sweden (ca 1.25 g mycelial biomass per m^2^, June–August) (Clemmensen et al., 2006), as well as that recorded at the tree line in the Swiss Alps (ca 0.4–1.75 g mycelial biomass per m^2^, June‐September) (Hagedorn et al., 2013). In other northern biomes, Wallander, Göransson, and Rosengren (2004) reported hyphal biomass to be 29.5 and 21 g C/m^2^ in a Norway spruce forest and a mixed oak–spruce forest, respectively. If we compare these values to “aboveground” NPP, Berggren Kleja et al. (2008) reported NPP estimates ranging from 333 to 590 g C m^−2^ year^−1^ in three 40‐year‐old Swedish *Picea abies* forests, whereas in a mountain birch forest, the NPP is considerable lower (2.5 g C m^−2^ year^−1^; calculated from data given in Hedenås et al. (2011)). Hence, EMM production appears to account for a larger part of total ecosystem productivity in the mountain birch forest (approximately 25%) compared to a boreal forest (approximately 10%).

## 5 CONCLUDING REMARKS

This study, one of the first of its kind in arctic and alpine tree line ecosystems, shows that aboveground grazing affects EMM biomass in these environments. The EMM biomass in the birch forest at Fulufjället and Långfjället was positively affected by the exclusion of herbivores, as hypothesized. In contrast, grazer exclusion at the shrub heath at Långfjället resulted in reduced mycelial biomass. This surprising finding implies a large degree of context dependency in how EMM production changes under grazing. In view of recent studies that have highlighted the importance of ECM for decomposition of soil organic matter (Hartley et al., 2012), such a stimulation of EMM growth could potentially lead to an acceleration of C cycling in the subarctic. Despite being a crucial component in arctic C dynamics, the influence of herbivory on microbial processes is a vastly understudied area. Consequently, more studies, which address the effect of grazing on the complete mycorrhizal system (both root tip and EMM), are needed to further explore the interaction between herbivores, plant community change, and C dynamics in northern ecosystems.

## CONFLICT OF INTEREST

None declared.

## AUTHOR CONTRIBUTIONS

T.V. and R.G.B. designed the study and conducted the field work. F.L., A.E., B.F., M.R., and R.G.B. conducted the laboratory work. M.R. and B.F. planned the community sequencing. M.B. conducted bioinformatics analyses. T.V., F.L., M.B., and R.G.B analyzed the data. T.V. and F.L. led the writing of the manuscript with the help of R.G.B. All authors contributed critically to the drafts and gave final approval for publication.

## DATA ACCESSIBILITY

All consensus (read of insert) sequences were submitted to the Short Read Archive (SRA) under the accession number SRP120113.
