# Supplementary figures and images for: Complex effects of mammalian grazing on extramatrical mycelial biomass in the Scandes forest‐tundra ecotone
Source: Ecol Evol. 2017 Dec 14;8(2):1019–30. doi: 10.1002/ece3.3657 (PMC5773333; doi:10.1002/ece3.3657)

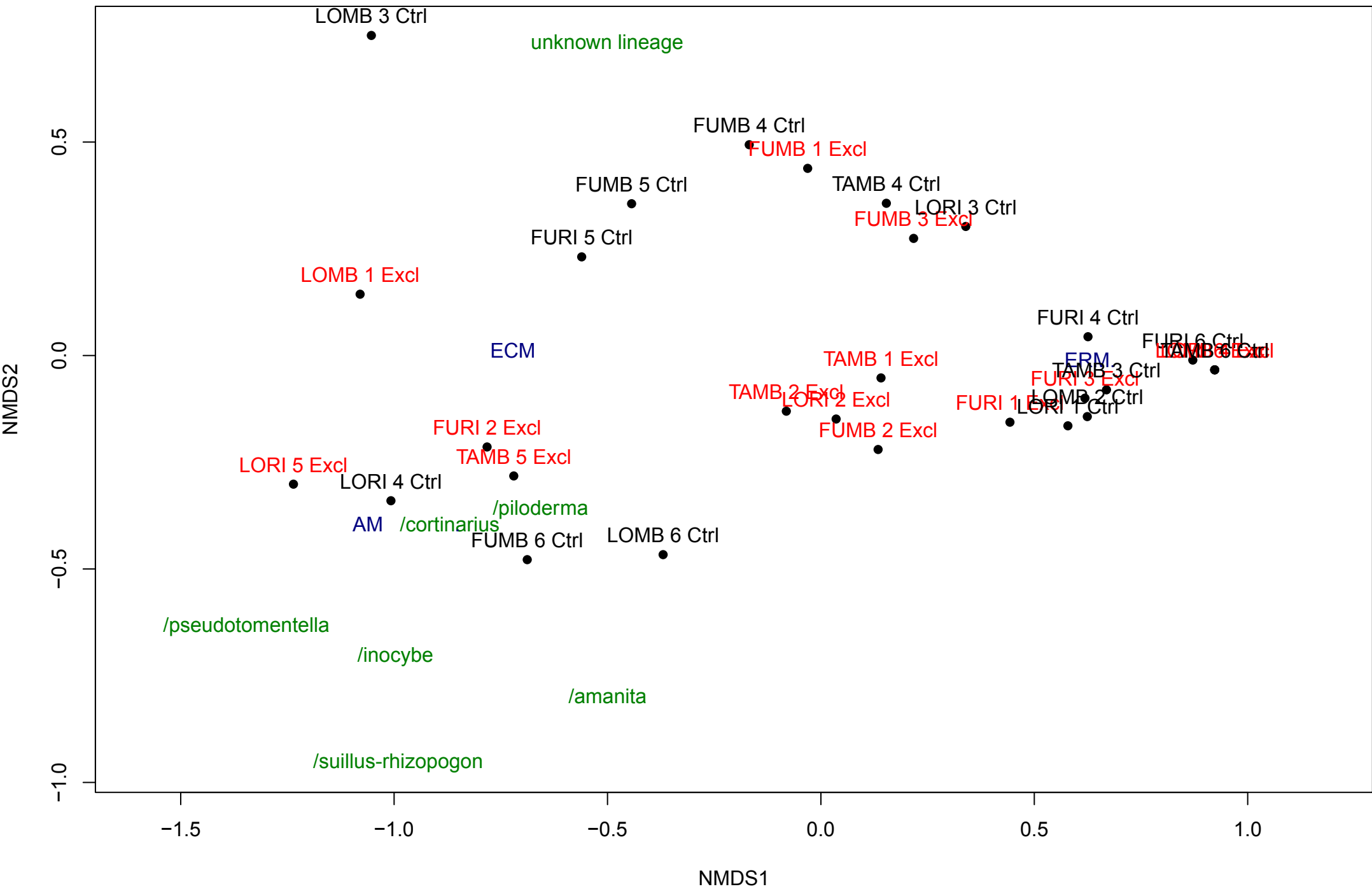

Supplement: Supplementary file 1 [file ECE3-8-1019-s001.pdf]
