# Supplementary material for: Complex effects of mammalian grazing on extramatrical mycelial biomass in the Scandes forest‐tundra ecotone
Source: Ecol Evol. 2017 Dec 14;8(2):1019–30. doi: 10.1002/ece3.3657 (PMC5773333; doi:10.1002/ece3.3657)
Supplement: Supplementary file 2 [file ECE3-8-1019-s002.docx]

**Table S1.** N and C content (%), C/N ratios, Soil organic matter (% SOM) and pH in mineral and humus soil.

|  |  |  | **Mineral soil** | | | | | | **Humus soil** | | | | | |
| --- | --- | --- | --- | --- | --- | --- | --- | --- | --- | --- | --- | --- | --- | --- |
| Veg type | Site | Treatm | N% | C% | C/N | SOM (%) | pH H2O | pH KCl | N% | C% | C/N | SOM (%) | pH H2O | pH KCl |
| **Shrub** | Fulu | Amb | 0.3±0.1 | 7.1±2.1 | 26.9±3.0 | 10.0±2.5 | 4.9±0.1 | 3.5±0.1 | 0.9±0.0 | 24.0±1.6 | 27.6±1.2 | 46.9±3.4 | 4.5±0.0 | 3.3±0.1 |
| **heath** |  | Excl | 0.5±0.1 | 14.3±1.6 | 28.6±1.6 | 28.5±6.6 | 4.3±0.0 | 3.3±0.0 | 1.4±0.1 | 40.8±0.9 | 29.4±0.9 | 79.2±6.1 | 4.2±0.2 | 3.2±0.0 |
|  | Lång | Amb | 0.1±0.0 | 2.0±0.5 | 29.2±2.8 | 4.4±0.7 | 5.1±0.0 | 3.5±0.0 | 0.7±0.0 | 22.7±1.2 | 30.8±1.4 | 45.3±1.5 | 4.6±0.1 | 3.3±0.0 |
|  |  | Excl | 0.1±0.0 | 2.5±0.7 | 26.7±3.0 | 4.5±1.5 | 4.9±0.2 | 3.3±0.1 | 0.9±0.1 | 26.6±2.3 | 29.7±1.7 | 46.3±5.7 | 4.8±0.1 | 3.3±0.0 |
| **Birch** | Fulu | Amb | 0.4±0.1 | 11.6±1.8 | 34.6±4.8 | 14.4±6.1 | 4.7±0.3 | 3.8±0.2 | 1.4±0.1 | 35.6±3.7 | 25.9±0.9 | 72.9±5.9 | 4.3±0.1 | 3.5±0.1 |
| **forest** |  | Excl | 0.3±0.1 | 6.8±1.6 | 25.1±1.3 | 9.9±2.4 | 4.5±0.2 | 3.6±0.1 | 1.3±0.1 | 32.6±1.0 | 24.9±1.2 | 66.8±2.9 | 4.4±0.2 | 3.5±0.1 |
|  | Lång | Amb | 0.2±0.1 | 3.7±1.5 | 21.7±2.7 | 6.5±2.2 | 4.6±0.2 | 3.7±0.2 | 0.9±0.3 | 23.7±7.9 | 24.6±2.9 | 44.0±15.8 | 4.5±0.5 | 3.7±0.6 |
|  |  | Excl | 0.2±0.1 | 5.1±1.6 | 25.2±1.7 | 7.6±1.0 | 4.5±0.2 | 3.4±0.1 | 0.9±0.1 | 24.4±1.3 | 28.6±2.3 | 48.5±2.6 | 4.1±0.2 | 3.4±0.2 |
|  | Pulsu | Amb | 0.1±0.0 | 3.0±0.3 | 23.6±1.0 | 6.6±0.6 | 4.8±0.0 | 3.7±0.0 | 0.8±0.0 | 21.9±1.7 | 27.0±2.0 | 46.8±1.8 | 4.1±0.1 | 3.6±0.1 |
|  |  | Excl | 0.1±0.0 | 4.2±0.5 | 29.5±2.8 | 8.0±0.7 | 4.7±0.2 | 3.7±0.1 | 0.8±0.1 | 22.8±1.7 | 30.0±2.1 | 43.2±3.7 | 4.1±0.1 | 3.6±0.2 |

**Table S2.** N and C content (%) and C/N ratios in mineral soil roots and humus soil roots.

|  |  | **Mineral soil roots** | | | **Humus soil roots** | | |
| --- | --- | --- | --- | --- | --- | --- | --- |
| Veg type | Site | N% | C% | C/N | N% | C% | C/N |
| **Shrub** | Fulu | 0.5±0.0 | 41.7±0.8 | 88.1±3.4 | 0.6±0.0 | 45.4±0.7 | 71.4±3.1 |
| **heath** |  | 0.7±0.0 | 45.2±1.1 | 64.6±4.9 | 0.6±0.0 | 44.7±0.1 | 78.5±3.0 |
|  | Lång | 0.4±0.0 | 44.3±0.5 | 101.0±10.3 | 0.6±0.1 | 45.9±1.9 | 84.8±11.1 |
|  |  | 0.5±0.0 | 42.8±1.5 | 85.3±3.4 | 0.6±0.0 | 46.2±1.1 | 83.0±4.9 |
| **Birch** | Fulu | 0.6±0.0 | 42.0±2.3 | 65.7±3.0 | 0.7±0.0 | 47.4±0.2 | 69.0±2.6 |
| **forest** |  | 0.6±0.0 | 39.1±2.2 | 62.2±6.1 | 0.6±0.0 | 46.5±0.2 | 74.0±1.9 |
|  | Lång | 0.6±0.1 | 41.8±1.7 | 69.5±11.8 | 0.8±0.2 | 45.1±1.3 | 59.5±11.7 |
|  |  | 0.7±0.0 | 43.5±3.0 | 63.2±3.1 | 0.7±0.0 | 45.0±0.5 | 61.0±2.7 |
|  | Pulsu | 0.7±0.1 | 44.0±0.8 | 66.9±5.2 | 0.8±0.1 | 45.4±0.6 | 61.4±5.7 |
|  |  | 0.6±0.0 | 42.9±1.5 | 71.0±4.5 | 0.7±0.1 | 45.5±1.0 | 68.4±6.3 |
